# Sexual Dysfunction and Hyperprolactinemia in Male Psychotic Inpatients: A Cross-Sectional Study

**DOI:** 10.1155/2011/686924

**Published:** 2011-11-30

**Authors:** Erik Johnsen, Rune Kroken, Else-Marie Løberg, Eirik Kjelby, Hugo A. Jørgensen

**Affiliations:** ^1^Division of Psychiatry, Haukeland University Hospital, Sandviksleitet 1, 5035 Bergen, Norway; ^2^Department of Clinical Medicine, Psychiatry, University of Bergen, Sandviksleitet 1, 5035 Bergen, Norway; ^3^Institute Biological and Medical Psychology, University of Bergen, Sandviksleitet 1, 5035 Bergen, Norway

## Abstract

*Introduction*. Sexual dysfunction (SD) and hyperprolactinemia are frequently reported in patients with psychotic disorders and have the potential for severe complications but investigations in males are particularly scarce. The primary aims were to determine the prevalence of SD and hyperprolactinemia in male patients and to investigate whether associations exist between SD and prolactin levels. *Methods*. Cross-sectional data were obtained at discharge from the hospital or 6 weeks after admittance for patients acutely admitted for psychosis and treated with a second-generation antipsychotic drug. *Results*. Half the patients reported diminished sexual desire and more than a third reported erectile and ejaculatory dysfunctions with no differences among the drugs. More than half the sample was hyperprolactinemic. No association was found between prolactin levels and SD. *Conclusion*. High rates of SD and hyperprolactinemia were found in male patients and should be a treatment target. SD and hyperprolactinemia were not correlated.

## 1. Introduction

Active psychosis affects most aspects of normal functioning and has been ranked the third most disabling disorder in the general population, and more disabling than paraplegia, blindness, or HIV infection [1]. The life-time prevalence of any psychotic disorder is about 3 in 100 persons [2]. In a substantial proportion of cases, the disorders are chronic and life long. The presence of psychosis will in most instances indicate the use of antipsychotic drugs. Both the nature of the disorders and antipsychotic drug treatment can profoundly affect sexual functioning.

Main tolerability issues related to antipsychotic drug use have traditionally been the extrapyramidal syndrome (EPS) associated with the first-generation (typical) antipsychotics, and metabolic adverse effects associated mainly with the second-generation (atypical) agents [3, 4]. Sexual dysfunction (SD) has received far less attention, although these side effects have been reported among the most discomforting ones by patients with schizophrenia [5, 6]. SD is important also as it has negative impact on medication adherence. Antipsychotic-induced hyperprolactinemia is commonly regarded as a frequent cause of SD. As demonstrated in a review by Byerly et al. [7], the findings of different studies are conflicting, however, with regards to associations between hyperprolactinemia and sexual side effects. While differential propensities among second-generation antipsychotics (SGAs) in causing hyperprolactinemia are well documented [8], differences among the SGAs in causing SD are less investigated. Studies addressing male SD specifically in psychosis are particularly scarce. In one study, SD has been reported to affect almost half the sample of outpatients with schizophrenia and to adversely affect their quality of life [9].

Several research questions of clinical relevance thus remain unresolved, and studies in clinically relevant samples are called for.

The primary aims of the present study were to determine the prevalence of SD and hyperprolactinemia, and to investigate whether associations exist between SD and prolactin levels in male patients with psychosis. Secondary aims were to disclose whether differences exist among second-generation antipsychotics (SGA) with regards to SD.

## 2. Materials and Methods

The materials and methods used have been described in more detail in a previous publication [10]. The Bergen psychosis project (BPP) is a pragmatic, randomized trial comparing SGAs in the treatment of psychosis. The present study reports results from the BPP from the time of discharge or 6 weeks after admission if not discharged from hospital. To ensure a clinically relevant sample, the patient recruitment focused on all patients with psychosis acutely admitted to the emergency ward. Patients were recruited from March 2004 until February 2009. All patients were recruited from Haukeland University Hospital, Division of Psychiatry, with a catchment population of about 400,000. The BPP was approved by the Regional Committee for Medical Research Ethics, and the Norwegian Social Science Data Services. The BPP was publicly funded and has not received any financial or other support from the pharmaceutical industry.

The Regional Committee for Medical Research Ethics allowed eligible patients to be included before informed consent was provided, thus entailing a clinically relevant representation in the study. Patients from 18 to 65 years of age were eligible for the study if they were acutely admitted to the emergency ward for symptoms of psychosis as determined by a score of ≥4 on one or more of the items delusions, hallucinatory behavior, grandiosity, suspiciousness/persecution, or unusual thought content from the Positive and Negative Syndrome Scale (PANSS) [11]. Eligible patients met the ICD-10 [12] diagnostic criteria for schizophrenia, schizoaffective disorder, acute and transient psychotic disorder, delusional disorder, drug-induced psychosis, bipolar disorder except manic psychosis, and major depressive disorder with psychotic features. The diagnoses were determined by the hospital's psychiatrists or specialists in clinical psychology. Patients were excluded from the study if they were unable to use oral antipsychotics because a depot formulation was indicated, were suffering from manic psychosis or for other behavioral or mental reasons related to the state of illness were unable to cooperate with assessments, did not understand spoken Norwegian, were candidates for electroconvulsive therapy as determined by the attending psychiatrists, or were medicated with clozapine, usually regarded as a final resort, on admittance. Patients with drug-induced psychoses were included only when the condition did not resolve within a few days and when antipsychotic drug therapy was indicated.

The patients were rated using the PANSS, the Calgary Depression Scale for Schizophrenia (CDSS) [13], the Clinical Drug and Alcohol Use Scales (CDUS/CAUS) [14], the Clinical Global Impression—Severity of Illness scale (CGI-S) [15], the Global Assessment of Functioning—Split Version, Functions scale (GAF-F) [16], and a neurocognitive screening test (Repeatable Battery for the Assessment of Neuropsychological Status (RBANS)) [17]. Patients were asked also to complete the patient-administered version of the UKU Side Effect Rating Scale (UKU-SERS Pat) [18]. The items reported here include diminished sexual desire; erectile dysfunction; and ejaculatory dysfunction. The patient-administered version of the interview was chosen to obtain more valid results as clinicians often underestimate SD [19]. The questions asked were “have you experienced decreased sexual interest or decreased sexual desire?”; “have you experienced difficulty in reaching erection?”; “have you experienced difficulties in ejaculation?” The patients were instructed to report symptoms that they attributed to their prescribed medications and use the last week as the time frame of reference. Each item was rated from 0 (not at all) to 3 with increasing severity of the SD symptom reported. A composite mean SD score was calculated based on the three UKU-SERS Pat items, accepting up to two missing values.

A blood sample was collected from the patients between 08 and 10 am, and serum level measurements of prolactin and antipsychotics were conducted. Drug doses were converted to mean Defined Daily Doses (DDDs) as developed by the World Health Organization Collaborating Centre for Drug Statistics Methodology [20]. The basic definition of the DDD unit is the assumed average maintenance dose per day for a drug used for its main indication in adults. Fifteen (23.1%) of the prolactin blood samples were analysed at Laboratory A by means of a noncompetitive immunofluorometric assay (DELFIA kit by Wallac Oy, Turku, Finland). Fifty (76.9%) of the samples were analyzed at Laboratory B using another immunoassay kit (Immulite 2000 by Siemens Medical Solutions Diagnostics, Berlin and Munich, Germany). The cutoff for hyperprolactinemia was set at 360 mIU/L. Screening for macroprolactinemia by polyethylenglycol (PEG) precipitation was performed if prolactin levels were above 1000 mIU/L at both laboratories to identify cases with pseudohyperprolactinemia caused by the biologically inert macroprolactin fraction.

SPSS version 18.0 was used for statistical analyses. Mean serum prolactin levels at laboratories A and B were compared using an independent samples *t*-test. Chi square exact test was used for categorical data. To investigate the association between prolactin levels and symptoms registered on the rating scale, a bivariate analysis of correlation was performed using the Spearman correlation coefficient as normal distribution could not be assumed. Significance level was set at *α* = 0.05.

## 3. Results and Discussion

### 3.1. Results

A total of 72 men were assessed. A total of 20 patients used risperidone, the corresponding figures were for olanzapine 26, quetiapine 9, ziprasidone 13, and aripiprazole 1. Three patients were not prescribed antipsychotics. The mean serum levels with standard deviations (sd) and reference levels in nanomoles per litre were for risperidone 79.5 (58.5) (30–120), for olanzapine 107.3 (75.8) (30–200), for quetiapine 522.8 (660.9) (100–800), for ziprasidone 129.1 (107.2) (30–200), and for aripiprazole 141 (-) (200–1300). The mean doses in milligrams with sd were for risperidone 3.7 (1.3), for olanzapine 17.3 (6.4), for quetiapine 477.8 (204.8), for ziprasidone 98.3 (46.3); and for aripiprazole 5 (-). There were no differences among the groups with regards to the use of concomitant medication.

**Figure 1:**
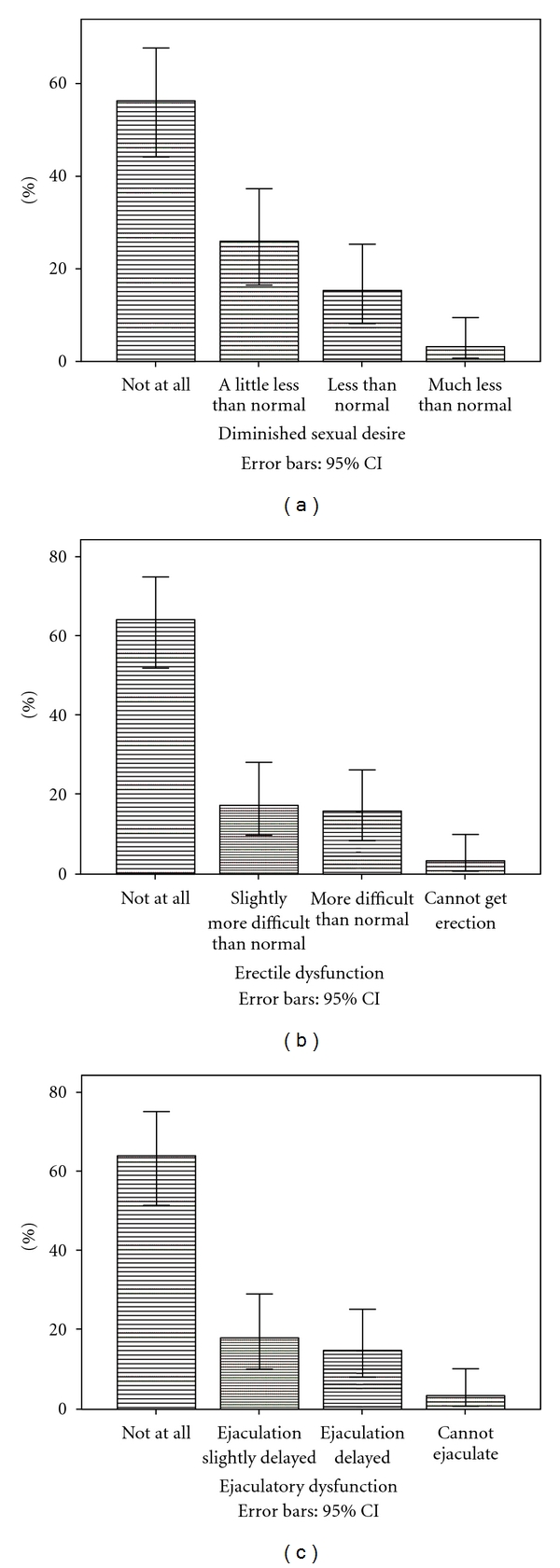
Distribution of sexual dysfunction.

**Figure 2:**
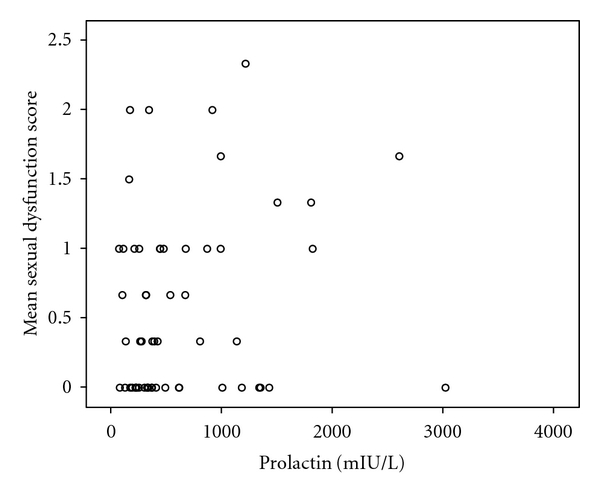
Sexual dysfunction and serum prolactin levels. Notes. Mean sexual dysfunction score = Composite mean sexual dysfunction score calculated based on the UKU-SERS Pat items Diminished sexual desire; Erectile dysfunction; and Ejaculatory dysfunction.

The clinical characteristics and demographics are displayed in Table 1. The majority had a diagnosis of schizophrenia (55.6%). A total of 33 (45.8%) patients had not used antipsychotic drugs before this admittance to hospital. With the exception of a higher CDSS sum score (6.9), in the risperidone group versus 2.8; 3.9; 3.6; 1.0; 3.5, in the olanzapine, quetiapine, ziprasidone, aripiprazole, and unmedicated groups, respectively (one-way anova, *P* = 0.033), there were no statistically significant differences among the drug groups. A total of 45.9% of the patients reported diminished sexual desire, whereas 35.9% and 36.1% reported erectile and ejaculatory dysfunction, respectively (Figure 1). There were no differences among the groups, or between the antipsychotic naïve patients and those with prior antipsychotic drug use in this regard. The mean prolactin level was 627.9 mIU/L, range 59.0–3019.0. There was no significant difference among the laboratories with regards to mean prolactin levels (*t*-test: *P* = 0.25; mean difference 68.3; 95% CI −286.0–422.6). The risperidone group had the highest mean prolactin level (1250.8 mIU/L) followed by olanzapine (483.0 mIU/L), ziprasidone (379.6 mIU/L), quetiapine (236.2 mIU/L), the unmedicated group (184.7 mIU/L), and aripiprazole (70.0 mIU/L, one-way anova: *P* < 0.001). A total of 56.3% of the patients had hyperprolactinemia, and there were significant differences among the groups following the same pattern as for the mean prolactin levels (chisquare: *P* < 0.001). A total of 18.5% had prolactin levels above 1080 mIU/L, the proportion being 57.9% in the risperidone group with significant differences among the groups (chisquare: *P* < 0.001). There was no association between prolactin level and the composite SD score (Spearman's correlation coefficient *r* = 0.141; *P* = 0.29, Figure 2). Also, there was no association between SD and the PANSS total and subscale scores; the CDSS, the CGI, the GAF-F, neurocognitive test score, or DDDs of the different drugs. For risperidone but not the other antipsychotics, there was a significant correlation between serum prolactin level and drug doses of risperidone in DDD equivalents (Spearman's correlation coefficient *r* = 0.598; *P* = 0.011).

### 3.2. Discussion

The sample was heterogeneous both with regards to diagnoses and stage of illness and about half the sample was antipsychotic drug naïve at admittance which most likely represents patients with first-episode psychosis. The sample should accordingly be clinically relevant. The main findings of the present study were the very high rates of SD and hyperprolactinemia in patients treated with SGAs, and the lack of association between the two. About half the patients reported diminished sexual desire and more than a third reported erectile and ejaculatory dysfunction. The rate of SD is in correspondence with the findings of Olfson et al. [9] in their sample of male outpatients with schizophrenia. The mean age of the sample was more than 10 years older than in the BPP sample and seemed to be selected for the assessment of SD specifically, making the results of the more diagnostically and clinically heterogeneous BPP sample even more startling. SD is perceived by patients as among the more severe side effects of antipsychotics and is associated with poor drug adherence [5, 6]. Noncompliance is one leading cause of relapse and rehospitalisation in patients with schizophrenia, the latter representing the largest part of the schizophrenia treatment costs [21]. The proportions with SD are accordingly alarmingly high both in terms of individual suffering and economic burden to society. With regards to the secondary aim, no differences among the drug groups were found. Bobes et al. [22] found in their sample of 636 schizophrenia outpatients a lower risk of SD in quetiapine, treated patients compared to those treated with haloperidol, risperidone, or olanzapine. The different findings across the studies may be related to different samples, treatment settings, or an insufficient sample size in the BPP to detect actual differences among the drug groups.

More than half the sample was hyperprolactinemic, and about one fifth had prolactin levels more than 3 times the upper threshold, none of which were caused by the biologically inert macroprolactin fraction. There were differences among the drug groups, with risperidone-treated patients having the highest prolactin levels and the highest proportions with hyperprolactinemia. Hyperprolactinemia has received new attention lately as potential long-term complications have been pointed to, including osteoporosis and carcinogenic effects [7, 8]. No association was found in the present study between prolactin levels and SD. This is in line with previous findings from our research group [23]. In a recent study in schizophrenia patients switched to a second-generation antipsychotic drug, positive correlation between prolactin levels and diminished sexual desire was found for men [24]. Nakonezny et al. [25] found an association between serum prolactin level and SD for prolactin but not quetiapine in their 6-week randomized trial. Byerly et al. [7] report differing results among different studies regarding relationship between prolactin levels and SD in men, as only about half the studies reviewed found support for such a relationship. Data on SD in first-episode psychosis has been published from the EUFEST study, indicating influence from the psychotic disorder itself as well as from prolactin on at least some aspects of SD [26].

The primary strengths of the present study are the clinically relevant sample of consecutively recruited male psychotic patients, and the very comprehensive characterisation of the sample. The measurement of serum levels of the antipsychotics used adds special value to the study too.

Some limitations should be mentioned, however. The cross-sectional design does not allow for analyses of causality. The sample size may have been too small to detect actual associations. We do not believe this to be the case, however, as significant correlations between hyperprolactinemia and SD were disclosed in even smaller samples of schizophrenia patients in the review by Byerly et al. [7]. We hypothesise that in a heterogeneous sample such as the BPP sample, the prolactin contribution is blurred among several other causes of SD.

## 4. Conclusions

Both SD and hyperprolactinemia were very prevalent in this sample of male psychotic patients. Based on our findings, the phenomena should be regarded as relatively independent entities with regards to planning appropriate actions. In some instances, reduction of the prolactin level may also resolve SD, whereas in other cases this may not suffice. Finally, prolactin levels should be measured irrespective of whether symptoms of SD are present or not to avoid potential long-term complications of “silent” hyperprolactinemia.

## Figures and Tables

**Table 1:** Clinical and demographic characteristics.

	Risperidone *N* = 20	Olanzapine *N* = 26	Quetiapine *N* = 9	Ziprasidone *N* = 13	Aripiprazole *N* = 1	Unmedicated *N* = 3	Total *N* = 72
	Mean (SD)	Mean (SD)	Mean (SD)	Mean (SD)	Mean (SD)	Mean (SD)	Mean (SD)
Age (years)	31.9 (13.4)	30.0 (8.8)	39.2 (12.7)	36.0 (16.6)	33.0	29.7 (4.7)	32.8 (12.3)
PANSS positive score	13.8 (3.6)	13.3 (4.4)	11.3 (3.9)	12.2 (3.7)	9.0	16.0 (6.1)	13.0 (4.1)
PANSS negative score	17.6 (6.7)	15.2 (6.6)	12.6 (5.3)	15.2 (5.9)	24.0	17.7 (7.6)	15.8 (6.5)
PANSS general score	28.2 (6.4)	24.7 (5.8)	23.7 (5.0)	25.2 (4.1)	32.0	28.0 (5.3)	25.9 (5.7)
PANSS total score	59.5 (13.5)	53.2 (13.2)	47.6 (12.5)	52.6 (10.4)	65.0	61.7 (18.8)	54.7 (13.2)
CDSS score	6.9 (4.8)	2.8 (3.4)	3.9 (3.9)	3.6 (3.7)	1.0	3.5 (2.1)	4.2 (4.2)*
GAF-F score	38.9 (8.7)	39.3 (7.9)	38.0 (5.5)	39.9 (7.7)	44.0	35.7 (4.0)	39.0 (7.6)
CGI-S score	4.0 (0.9)	3.5 (1.0)	3.0 (1.0)	3.7 (1.1)	4.0	4.0 (1.0)	3.63 (1.0)
Neurocogn. *t*-score	41.7 (8.0)	39.8 (7.7)	42.8 (5.8)	40.3 (8.3)	47.8	38.4 (-)	41.0 (7.5)
	*N* (%)	*N* (%)	*N* (%)	*N* (%)	*N* (%)	*N* (%)	*N* (%)
Ethnicity-whites	17 (85.0)	22 (84.6)	7 (77.8)	13 (100.0)	1 (100.0)	3 (100.0)	63 (87.5)
Drug naive	7 (35.0)	14 (53.8)	5 (55.6)	7 (53.8)	0	0	33 (45.8)
Illicit drug abuse^1^	5 (25.0)	6 (23.1)	1 (11.1)	0	0	2 (66.7)	14 (19.4)
Alcohol abuse^1^	2 (11.1)	2 (7.7)	1 (11.1)	1 (7.7)	0	0	6 (8.3)

Diagnoses							

Drug-induced psychosis	3 (15.0)	2 (7.7)	0	1 (7.7)	0	1 (33.3)	7 (9.7)
Schizophrenia	10 (50.0)	16 (61.5)	5 (55.6)	7 (53.8)	1 (100.0)	1 (33.3)	40 (55.6)
Acute	1 (5.0)	3 (11.5)	1 (11.1)	0	0	1 (33.3)	6 (8.3)
Affective	4 (20.0)	3 (11.5)	1 (11.1)	3 (23.1)	0	0	11 (15.3)
Psychosis unspec.	0	2 (7.7)	1 (11.1)	0	0	0	3 (4.2)
Other/missing	2 (10.0)	0	1 (11.1)	2 (15.4)	0	0	5 (6.9)

Notes: *N* = number of patients; SD = standard deviation (omitted in the aripiprazole group as only one patient); antipsychotic drug naïve = no life-time exposure to antipsychotic drugs before study inclusion; Schizophrenia = schizophrenia, schizo-affective disorder, acute polymorphic psychotic disorder with symptoms of schizophrenia, acute schizophrenia-like psychotic disorder, delusional disorder; Acute = acute psychosis other than those categorized under Schizophrenia; Affective = affective psychosis; Psychosis unspecified = miscellaneous psychotic disorders. All diagnoses are according to ICD-10; PANSS = the positive and Negative Syndrome Scale; CDSS = the Calgary Depression Scale for Schizophrenia; GAF-F = the Global Assessment of Functioning, split version, Functions scale; CGI = the Clinical Global Impression, severity of illness scale; Neurocogn. = sum *t*-score of the Repeatable Battery for the Assessment of Neuropsychological Status. ^1^Last 6 months; *P < 0.05.
